# Andean *Prumnopitys Andina* (Podocarpacae) Fruit Extracts: Characterization of Secondary Metabolites and Potential Cytoprotective Effect

**DOI:** 10.3390/molecules24224028

**Published:** 2019-11-07

**Authors:** Felipe Jiménez-Aspee, Cristina Theoduloz, Lisa Pormetter, Judith Mettke, Felipe Ávila, Guillermo Schmeda-Hirschmann

**Affiliations:** 1 Departamento de Ciencias Básicas Biomédicas, Facultad de Ciencias de la Salud, Universidad de Talca, Talca 3460000, Chile; fjimenez@utalca.cl; 2 Laboratorio de Cultivo Celular, Facultad de Ciencias de la Salud, Universidad de Talca, Talca 3460000, Chile; 3 Laboratorio de Química de Productos Naturales, Instituto de Química de Recursos Naturales, Universidad de Talca, Talca 3460000, Chile; lisa.p@alice-dsl.net (L.P.); j.mettke@gmx.de (J.M.); schmeda@utalca.cl (G.S.-H.); 4 Escuela de Nutrición y Dietética, Facultad de Ciencias de la Salud, Universidad de Talca, Talca 3460000, Chile; favilac@utalca.cl

**Keywords:** *Prumnopitys andina*, counter-current chromatography, 20-hydroxyecdysone, high-performance liquid chromatography, cell-based assays, thiobarbituric acid reactive species

## Abstract

The fruits from the Chilean Podocarpaceae *Prumnopitys andina* have been consumed since pre-Hispanic times. Little is known about the composition and biological properties of this fruit. The aim of this work was to identify the secondary metabolites of the edible part of *P. andina* fruits and to assess their antioxidant activity by means of chemical and cell-based assays. Methanol extracts from *P. andina* fruits were fractionated on a XAD7 resin and the main compounds were isolated by chromatographic means. Antioxidant activity was determined by means of 2,2-diphenyl-1-picrylhydrazyl radical (DPPH), ferric reducing power (FRAP), trolox equivalent antioxidant capacity (TEAC) and oxygen radical absorbance capacity (ORAC) assays. The cytoprotective activity of the extract against oxidative and dicarbonyl stress was evaluated in human gastric epithelial cells (AGS). The total intracellular antioxidant activity (TAA) of the extract was determined in AGS cells. The inhibition of meat lipoperoxidation was evaluated under simulated gastric digestion conditions. Rutin, caffeic acid β-glucoside and 20-hydroxyecdysone were identified as major components of the fruit extract. Additional compounds were identified by high-performance liquid chromatography diode-array detector mass spectrometry (HPLC-DAD-MS^n^) and/or co-injection with standards. Extracts showed dose-dependent cytoprotective effects against oxidative and dicarbonyl-induced damage in AGS cells. The TAA increased with the pre-incubation of AGS cells with the extract. This is the first report on the composition and biological activity of this Andean fruit.

## 1. Introduction

The Podocarpaceae are gymnosperms occurring mainly in the Southern Hemisphere and comprise 20 genera and about 190 species [1]. Most studies on this plant family have been carried out on the genus *Podocarpus*, including species from Australia, Asia and South Africa [2], with a focus on constituents from the bark, stem and leaves. The arils from the Australian and New Zealand species *Podocarpus dacrydioides*, *P. neriifolius*, *P. nivalis*, *P. salignus* and *P. totara* are eaten raw or cooked. In Australia, the fruits from *P. spinulosus* are used locally to produce jams and preserves while the fruits from *P. nagi* are consumed in the Himalaya mountain ranges. The fleshy stem of *Podocarpus elatus* is eaten by the Australian Aborigine and the fruits are consumed raw or cooked [2].

**Figure 1:**
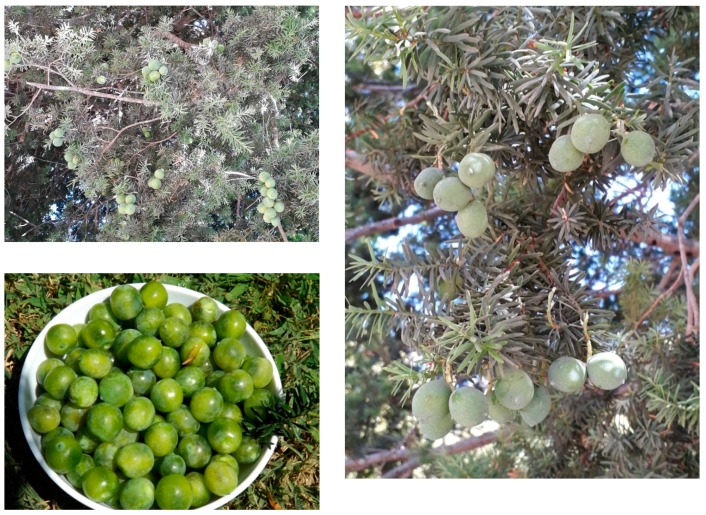
Fruiting *Prumnpitys andina* tree and detail of the ripe fruits, known as “Andean grapes”.

The sweet fruits from the Chilean native Podocarpaceae *Prumnopitys andina* Poepp. et Endl. de Laub., known in Chile as “lleuque” or “uva de cordillera” (Andean grape) have been appreciated as food since pre-Hispanic times, eaten raw and later processed into jam and preserves. The ripe fruits of *P. andina* are about 20 mm long and 15 mm wide, with an average weight of 3.6 g, bright-green in colour and contain one seed (Figure 1). The fleshy aril (edible part) corresponds to 70% of the fresh weight. This tree grows in the western Andean slopes of central Chile, from the Province of Linares to the Province of Cautín [3]. The populations of this tree are decreasing due to deforestation and overgrazing [4].

Previous studies on *P. andina* have been focused on diterpenes and phenolics from the bark and leaves. The tree bark is a good source of the diterpene ferruginol and other abietane diterpenes with demonstrated gastroprotective activity in rodent models [5] and antibacterial effects [6]. Less is known about the composition, chemistry and potential bioactivity of edible *Podocarpus* fruits. Aril extracts from *P. elatus* containing cyanidin-3-glucoside and pelargonidin-3-glucoside showed higher antioxidant capacity than blueberries in DPPH and FRAP assays [7,8]. However, a methanol (MeOH) extract from *P. elatus* did not protect RAW 264.7 cells from oxidative stress [9]. The methanol extract from *P. macrophyllus* presented moderate inhibitory activity towards α-glucosidase and scavenging capacity of the DPPH radical [10]. No components were identified in this study. To the best of our knowledge, there is no information about the chemistry of *P. andina* fruit extracts in mainstream scientific literature.

This study aimed to isolate and identify the main secondary metabolites from *P. andina* fruit arils, characterize their radical scavenging activity in chemical screening assays, their potential antioxidant activity in cell-based assays (human AGS gastric adenocarcinoma cells), and their potential ability to inhibit carbohydrate and lipid metabolizing enzymes and lipoperoxidation of meat during digestion under in vitro model conditions.

## 2. Results and Discussion 

### 2.1. Isolation of Main Constituents from the Fruit Extract

**Figure 2:**
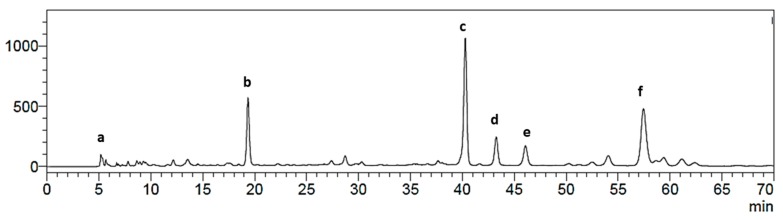
High-performance liquid chromatography diode-array detector (HPLC-DAD) chromatogram of the XAD-7 enriched fraction of the *P. andina* arils. Compounds (**a**) caffeic acid β-glucoside; (**b**) 3-O-caffeoylquinic acid; (**c**) 20-hydroxyecdysone; (**d**) orientin; (**e**) quercetin-3-O- β-rutinoside (rutin); (**f**) quercetin-3-O-β-D-acetyl glucoside.

The high-performance liquid chromatography diode-array detector (HPLC-DAD) chromatogram of the *P. andina* aril extract retained in the resin Amberlite XAD7 showed a fingerprint with a main compound eluting at Rt 40.4 min and showing ultraviolet (UV) maxima at 249 nm and three phenolic constituents with Rt of 19.7, 43.5, 46.5 and 58 min, respectively (Figure 2). The main constituents of the extract were isolated by Sephadex LH-20 gel permeation, followed by counter-current chromatography (CCC). The Sephadex fractions were combined based on the thin layer chromatographic (TLC) analysis as follows. Fractions 1–6 (1320 mg) showed polar polymeric compounds remaining at the origin on TLC analysis and were not further studied. 

The combined fractions 7–11 (479 mg) showed by TLC a major compound with Rf 0.43 and three minor spots with Rf values of 0.13, 0.25 and 0.94, respectively. The HPLC-DAD analysis of the mixture showed four main peaks with UV maxima at 254 nm and retention times of 19.7, 27.7, 38.2 and 40.4 min. The pooled fractions were further purified by CCC. This technique has been used in the past years for the fractionation and purification of numerous constituents present in food matrices [11]. The advantage of CCC, compared to Sephadex permeation and other column chromatography methods is the lack of a solid support, thus eliminating the risk of an irreversible adsorption of the extract in the stationary phase [12]. The CCC solvent system used in our experimental work was TBME-BuOH-ACN-H_2_O 3:1:1:5 *v/v*/*v/v*, showing K*_D_* values of 1.16, 1.79, 0.31 and 0.41. After an injection of 410 mg of the pooled fractions, a total of 90 fractions were collected at 2 min/tube (8 mL/tube). TLC analysis of the CCC separation showed that the compound with Rf = 0.43 (compound **c** in the HPLC fingerprint) was present in fractions 40–49. After lyophilisation, 72 mg of a colorless solid was obtained. The CCC fractions 1–39 and 50–90 showed minor components in insufficient amount to carry out further isolation. 

The infrared (IR) spectrum of the compound **c** showed an α, β-unsaturated carbonyl function at 1646 cm^−1^ and a broad hydroxyl band at 3404 cm^−1^. The ^1^H-nuclear magnetic resonance (NMR) spectrum showed three methyl singlets placed on sp^3^ oxygen-bearing C at δ 1.22, 1.22 and 1.21 ppm and two angular methyl singlets at δ 0.91 and 0.99 ppm, suggesting a steroid skeleton. A conjugated double bond at δ 5.83 and three secondary hydroxyl groups at δ 3.97, 3.87, and 3.35 ppm support a polyhydroxy derivative in agreement with the ^13^C-NMR spectrum that showed 27 C signals. Six of the C signals are clearly associated with hydroxyl functions, including three d at δ 79.63, 68.61, 67.00, and three singlets at δ 84.57, 77.34 and 70.60 ppm. The α, β-unsaturated carbonyl function was confirmed by the s at δ 205.06 s and the double bond C at δ 120.74 and 166.50 ppm, in agreement with a hydroxyecdysone derivative. The acetylation of the compound (acetic acid/pyridine) afforded a triacetate. The mass spectra showed a molecular formula of C_27_H_44_O_7_ for the steroid (calc. for 480.3087) and C_33_H_50_O_10_ for the triacetate. The spectroscopic and spectrometric data are in agreement with 20-hydroxyecdysone [13,14]. The NMR data of the natural product and the triacetate are summarized in Table 1. 20-hydroxyecdysone. Colourless solid, Fourier transform infrared (FT-IR) (KBr, film): 3404 (broad, OH), 2964, 2923, 1646 (C=O), 1375, 1060 cm^−1^; Q-TOF-MS: 480.2616 (12) (calculated for C_27_H_44_O_7_: 480.3087), 462.2761 (100), 444.3112 (73), 426.3464 (20), 370.4141 (8); [α]^20^_D_ −94.47° (MeOH; 0.374 g/100 mL). Triacetate: Calculated for C_33_H_50_O_10_: 606.3404.

The 20-hydroxyecdysone is an ecdysteroid known as β-ecdysone, ecdysterone, crustecdysterone, isoinokosterone and has been reported from *Podocarpus elatus* and other Podocarpaceae species [15]. It is widespread in many arthropod species and occurs in certain plant families such as Asteraceae, Caryophyllaceae and Polypodiaceae [16]. In plants, ecdysteroids constitute a defense mechanism against insects inducing the molting process [16]. In mammals, the consumption of ecdysteroids has been related to hypoglycaemic, hypocholesterolemic and tissue growth-promoting activities [17]. Anabolic effects of 20-hydroxyecdysone has been reported in mammals at doses of 5 mg/kg/day [17]. 

The fractions 21–22 (33 mg) from the LH-20 column of the XAD-retained fraction of *P. andina* arils showed a main compound with Rf value of 0.44 (compound **a** from the HPLC fingerprint). The ^1^H-NMR spectrum showed d at δ 7.61 and 6.38 ppm (*J* = 16 Hz) indicating a *trans*-conjugated double bond as well as a tri-substituted aromatic ring at δ 7.54 (d, 1 Hz), 7.20 (dd, 8, 1 Hz) and 6.88 (d, 8 Hz) ppm, in agreement with caffeic acid. A d at δ 4.84 (*J* = 7.6 Hz) and sugar signals in the range of 3.42–3.96 ppm indicate a β-configurated sugar, identified as glucose by comparison with standards. The compound was identified as caffeic acid β-glucoside in agreement with the Q-TOF analysis that showed an exact mass of 342.0951 (calculated for C_15_H_18_O_9_). The content of this compound in the extracts was calculated using an external calibration curve with a caffeic acid standard. The content ranged between 9.37–68.08 mg caffeic acid equivalents/100 g XAD7-extract. In addition, the Q-TOF analysis of the minor constituents allowed the identification of caffeic acid pentoside [M−H]: 311.0744, calculated for C_14_H_16_O_8_: 312.0845; caffeic acid methyl ether pentoside [M−H]: 325.0832, calculated for C_15_H_18_O_8_: 326.1002 and caffeic acid dimethylether pentoside [M−H]: 339.0998, calculated for C_16_H_20_O_8_: 340.1158.

Caffeic acid β-glucoside. Yellowish powder, ^1^H-NMR (CD_3_OD): 8.55 s (1H); 7.61 d (16.0) (1H); 7.54 s (1H); 7.20 d (8.0) (1H), 6.88 d (8.0) (1H); 6.38 d (16.0) (1H); 4.84 d (7.6) (1H); 3.96 dd (12.0, 2.0) (1H); 3.80–3.87 m; 3.77 s (3H); 3.70–3.76 m (2H); 3.46–3.55 m; 3.42 t (8.8) (1H).

The fractions 31–32 from the LH-20 column (56 mg), contained a main compound with a Rf value of 0.27 (compound **f** from the HPLC fingerprint). The UV spectrum showed typical absorbance for a flavonol 3-*O*-glycoside with maxima at 352, 320 and 280 nm. The NMR spectra are in full agreement with quercetin 3-*O*-rutinoside (rutin). 

The identification was confirmed by Q-TOF analysis and co-chromatography with a rutin standard. The content of this compound in the extracts was calculated using an external calibration curve with a rutin standard. The content ranged between 1.12–8.99 mg rutin equivalents/100 g XAD7 extract. Compounds b, d and f were identified by UV-data and co-injection with standards as 3-*O*-caffeoylquinic acid (b), orientin (d) and quercetin-3-*O*-β-d-acetyl glucoside (f). The remaining fractions of the Sephadex column chromatography showed the presence of complex mixtures that were not possible to separate by CCC or other chromatographic techniques. Hence, the tentative identification of these minor compounds in the *P. andina* aril extract and Sephadex fractions was carried out by HPLC-DAD-MS^n^ using literature and authentic standards for comparison.

### 2.2. High-Performance Liquid Chromatography Diode-Array Detector Mass Spectrometry (HPLC-DAD-MS) Profile

**Figure 3:**
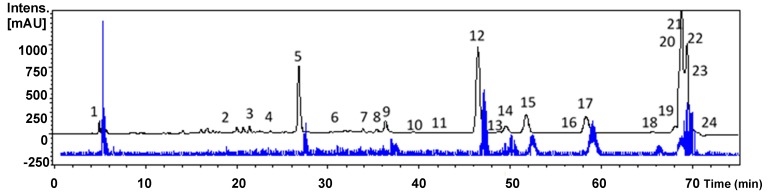
HPLC-DAD-MS/MS^n^ analysis of the extract from *Prumnopitys andina* fruits aril. Total ion chromatogram (TIC, blue) and ultraviolet (UV) chromatogram at 280 nm (black) of the extract. Numbers refer to Table 2.

The *P. andina* aril extract was analyzed by HPLC-DAD-MS^n^ to get a more complete insight into the fruit constituents. The HPLC-DAD-MS/MS^n^ analysis of the extract allowed the tentative identification of a series of constituents (mainly phenolics), based on the UV spectra, mass spectra and fragmentation patterns. Compounds are presented according to structural groups. A representative chromatogram is depicted in Figure 3. The compounds identified in the extract are summarized in Table 2. The description of the compounds tentatively identified is presented in the following paragraphs. 

#### 2.2.1. Simple Phenolics

Two benzoic acid hexosides bearing a hydroxy (compound **2**) or two hydroxy functions (compound **3**) were identified in the extract by the MS^2^ ion at 137 and 153 amu, after neutral loss of a hexose, in agreement with hydroxybenzoic acid hexoside (compound **2**) and dihydroxybenzoic acid hexoside (compound **3**), respectively.

#### 2.2.2. Phenylpropanoids

Several phenylpropanoids with different oxidation patterns in the aromatic ring were tentatively identified in the fruit. Caffeic acid hexosides (compounds **1** and **7**) were identified by the [M−H]^−^ ion at *m/z* 341, leading to *m/z* 179 amu after the neutral loss of a hexose. Compound **1** was identified as the isolated caffeic acid β-glucoside obtained from the Sephadex gel permeation chromatography. Three caffeoylquinic acid derivatives (compounds **4**, **5** and **8**) occur in the extracts and were assigned by comparison with the hierarchical scheme proposed by Clifford et al. [18] or co-injection with authentic standards. The compounds were assigned as 3,5-dicaffeoylquinic acid (compound **4**), 3-caffeoylquinic acid (compound **8**) and dicaffeoylquinic acid (compound **5**), respectively. The minor constituent 5-*p*-coumaroylquinic acid (compound **10**) was also identified by the fragmentation pattern, in agreement with Clifford et al. [18]. Hydroxymethoxycinnamic acid hexoside (compound **15**) was assigned by the [M−H]^−^ ion at *m/z* 355 amu, leading to the MS^2^ ion at *m/z* 193 amu after the neutral loss of an hexose, in agreement with the phenylpropanoid moiety. Compound **6**, showing a [M−H]^−^ ion at *m/z* 497 amu, leading to the MS^2^ ions at *m/z* 335 amu and 179 amu, is in agreement with caffeoylshikimic acid hexoside. 

#### 2.2.3. Flavonoids

The flavanonols dihydrokaempferol hexoside **9** and taxifolin hexoside **11** were identified based on the neutral loss of a hexose from the [M−H]^−^ ions, leading to the aglycone at *m/z* 287 amu for compound **9** and the aglycone after water loss for taxifolin (compound **11**), respectively [19]. The flavone C-glycoside orientin (compound **13**) was detected in the mixture, the fragmentation pattern is in agreement with literature [20]. The identity of this compound was confirmed by co-injection with a commercial standard. In addition, the compound **16** was identified as apigenin-C-hexoside by comparison with Geng et al. [21].

Flavonoids were identified on the basis of the extracted ion chromatograms of the aglycones, the neutral loss of sugars and esters as well as the UV spectrum of the main constituents. Most flavonoids were quercetin and kaempferol derivatives. The quercetin (Q) glycosides showed a MS2 base peak at m/z 301 amu and the neutral loss of rutinose (compound **17**, isolated from the Sephadex chromatography), hexose (compound **19**), pentose (compound **20**) and acetylhexose (compound **23**), in agreement with rutin, Q hexoside, Q pentoside and Q acetylhexoside, respectively. The exact position of the sugars remain to be confirmed. However, the UV spectra with maxima about 350 nm support the occurrence of Q-3-*O*-glycosides. 

The kaempferol glycosides showed the [M−H]^−^ ion of the aglycone at *m/z* 285 amu and were assigned as the dihexoside (compound **14**), the hexoside (compound **18**) and the rutinoside (compound **21**) based on the neutral loss of two hexose units, a hexose and a rutinose, respectively. Again, the UV spectra of the compounds detectable by the DAD showed maxima in agreement with 3-*O*-glycosides. The isorhamnetin rutinoside (compound **22**) and acetylhexoside (compound **24**) were assigned based on the neutral loss of rutinose and acetylhexose, respectively, leading to the [M−H]^−^ ion of the aglycone at *m/z* 315, in agreement with isorhamnetin/rhamnetin derivatives.

#### 2.2.4. Other Compounds

The isolated 20-hydroxyecdysone was detected in the HPLC-DAD-MSn as compound **12**, with a Rt of 46.1 min, with UV max at 249 nm. The compound showed a base peak ion at *m/z* 479 amu and secondary fragments at *m/z* 319 and 159 atomic mass units (amu). Considering that diterpenes have been reported in *Prumnopitys andina* bark and stems, we carried out an extracted ion chromatogram selectively looking for ferruginol and totarol (C_20_H_30_O, 286.45), abietic acid (C_20_H_30_O_2_, 302.45), dehydroabietic acid (C_20_H_28_O_2_, 300.44), hinokiol (C_20_H_30_O_2_, 302.46) and hinokione (C_20_H_28_O_2_, 300.44). The ionization mode selected did not allow the detection of these compounds. Further studies using other analytical approaches are needed to confirm their presence in the extracts from *P. andina* fruits.

### 2.3. Total Phenolics (TP) and Total Flavonoid (TF) Content

The total Folin-reactive material and total flavonoid content of methanol extracts *P. andina* fruits filtered on XAD7 are listed in Table 3. Levels were generally low, ranging from 0.3–1.1% w/fw. The TP content of *P. andina* fruit extracts ranged from 2.0–7.3 g gallic acid equivalents (GAE)/100 g XAD7 extract, while the TF content ranged from 1.3–5.3 g CE/100 g XAD7 extract (Table 3). The samples from 2017b and 2018 showed the highest TP and TF content. These values are consistent with TP content reported for other *Podocarpus* species: *P. elatus* fruits 1.16 g GAE/100 g fresh weight [7] and 7.64 g GAE/100 g dry weight [9]; *P. macrophyllus* fruit extract: 10.28 g GAE/100 g extract [10]. The total flavonoid content in stems and leaves from four African *Podocarpus* species ranged from 0.0013–0.0052 g CE/100 g dry weight [22]. However, it is important to note that Folin assays do not detect all classes of active compounds, including carotenoids and terpenes, such as 20-hydroxyecdysone. In addition, methanol does not extract all potentially active compounds, so the TP levels provide only one measure of aril phytocomposition.

### 2.4. Antioxidant Activity

Several methodologies based on different chemical mechanisms have been developed to determine the antioxidant capacity of a given sample. These methods provide different and complementary information about the interaction between radicals and samples. For an appropriate assessment of the antioxidant capacity of different food matrices, a single in vitro chemical method is not enough and should be complemented with other strategies [23]. In the present work, we evaluated the antioxidant activity of the samples using four different assays, including the scavenging of free radicals DPPH, ABTS (TEAC assay) and AAPH (ORAC assay), as well as the reducing power (FRAP assay). The antioxidant activity is summarized in Table 3. In all the determinations, the most active extract was the sample collected in 2018, except in the DPPH assay where the best antioxidant value was exhibited by sample 2017b. Interestingly, in the TEAC assay both samples collected in 2017 were inactive. In the ORAC assay the values ranged from 371.2–1921.2 µmol TE/g XAD7 extract. In the FRAP assay, the samples ranged from inactive to 473.3 µmol TE/g XAD7 extract. The 20-hydroxyecdysone was also evaluated in the antioxidant assays, proving to be inactive in the DPPH and TEAC assays. Interestingly, in the FRAP assay, this compound showed a value of 72.4 ± 7.9 µmol Trolox equivalents/g sample. In the ORAC assay, 20-hydroxyecdysone showed an experimental value of 827.3 ± 30.9 µmol TE/g of sample. Hence, the antioxidant capacity exhibited by the *P. andina* XAD7 extract might be attributed, at least in part, to 20-hydroxyecdysone. In a study carried out with Australian food plants, *Podocarpus elatus* (Illawarra plum) showed better antioxidant activity than blueberries in the DPPH and FRAP assays [7]. In another study, the *P. elatus* fruit extract also showed high antioxidant capacity in the TEAC assay [8]. 

In the ORAC assay, *P. elatus* fruit extract showed an ORAC value of 1111.1 µmol TE/g dry weight and a FRAP value of 864.2 µmol Fe2+/g dry weight [9]. *Podocarpus macrophyllus* showed similar IC_50_ values compared to our samples, with IC_50_ values of 65.6 µg/mL [10]. Compared to other Chilean native fruits, the extracts from *Prumnopitys andina* aril presented moderate to low antioxidant capacity [24,25]. It is important to note that the low antioxidant activity could be related to the lack of phenols and other compounds with known antioxidant capacity that were not extracted with methanol. In addition, the compounds present in the XAD7 extract could compete and counteract each other in the antioxidant assays. Future research should include the use of different solvents and separation procedures to examine the aril chemistry in a more detailed way.

### 2.5. Inhibition of Carbohydrate- and Lipid-Metabolizing Enzymes

In the α-glucosidase assay, the samples showed high inhibitory activities with IC_50_ values ranging between 4.6–8.7 µg/mL (Table 4). Under the same experimental conditions, the positive control acarbose presented an IC_50_ value of 137.7 µg/mL. The fruit extract from *Podocarpus macrophyllus* showed an IC_50_ value of 45.2 µg/mL against α-glucosidase [10]. It was recently reported that the extraction methodology, i.e., cold extraction vs. infusion, can influence the inhibitory activity of the extracts towards α-glucosidase [26].

Regarding the α-amylase and lipase inhibition assays, all samples proved to be devoid of activity, while positive controls acarbose and orlistat showed IC_50_ values of 28.5 and 0.04 µg/mL, respectively (Table 4). McDougall et al. [27] reported that fruit-extracts rich in tannins are more effective to inhibit α-amylase, while those richer in glycosylated flavonoids are prone to inhibit α-glucosidase. In a similar way, the inhibition of lipase has also been associated with higher content of tannins [28]. As described in Table 2, the *P. andina* fruit extracts does not present polymeric compounds. This could explain the absence of inhibition towards both enzymes.

The fractions obtained from the Sephadex chromatography were assessed as inhibitors of α-glucosidase to identify the most active fractions. The results showed that the inhibitory activity was concentrated among fractions 9–30, with the most active fractions being 13–20 (Table 5). The isolated compound caffeic acid, present in fractions 21–22, showed an IC_50_ value of 11.4 µg/mL, while rutin and 20-hydroxyecdysone only inhibited the enzyme by 44.8% and 40.1% at 50 µg/mL, respectively.

### 2.6. Determination of Thiobarbituric Acid Reactive Substances (TBARS) after Simulated Gastric Digestion

The consumption of a lipid-rich meal has been associated with an increase in post-prandial oxidative stress (PPOS) [29]. The PPOS comes along with inflammation, cellular oxidative stress and endothelial dysfunction [29]. According to Kanner et al. [30], lipoperoxidation and free radical generation are produced during food digestion in the stomach. The peroxidation of foods increases the amounts of malondialdehyde and other carbonyl compounds that can be absorbed in the small intestine and are associated with a higher risk of cardiovascular diseases [31]. Malondialdehyde (MDA) is one of the products formed through the decomposition of some primary and secondary lipid peroxidation products [32]. It is a highly toxic molecule and is usually used as a biological marker of oxidative stress [33]. It has been demonstrated that turkey meat digestion increases the MDA content and absorption levels in human volunteers, increasing the PPOS [30]. The simultaneous consumption of a polyphenol-rich food together with red or turkey meat prevents lipid peroxidation and MDA generation in the stomach. Moreover, this effect has been associated with a reduced PPOS [31]. A simulated gastric digestion model has been recently validated, by means of the thiobarbituric acid reactive substances (TBARS) method, to determine the extent of lipid peroxidation of several meat sources in the presence of polyphenols or other redox compounds from human diet. This model showed a good correlation with plasmatic MDA levels (determined by means of HPLC analysis) quantified in clinical trials (r^2^ = 0.91) [30]. The stomach presents the ideal conditions to promote MDA generation, due to the acid milieu and exposure to oxygen. However, a limitation of this method could be that it does not consider the presence of detoxifying enzymes present in the gastrointestinal tract such as glutathione-S-transferase [34].

The pooled samples of the *P. andina* fruit extract showed a high inhibitory effect towards the TBARS generation in turkey meat under simulated gastric digestion conditions. The IC_50_ value of the *P. andina* fruit extract was 95.0 ± 3.6 µg/mL, while the positive control aminoguanidine showed an IC_50_ value of 317.8 ± 20.4 µg/mL. Kanner et al. [30] showed that polyphenol-rich fruits such as blackberries, blackcurrants, raspberries, pomegranate, olives and plums among others, reduce the postprandial oxidative stress with IC_50_ values ranging from 2.0–13.0 g fruit/100 g turkey meat. Although the extracts from *P. andina* fruits do not have a high polyphenol content, our results show a TBARS inhibitory effect comparable to those exerted by the aforementioned polyphenol-rich fruits. 

### 2.7. Cell-Based Experiments

Human gastric epithelial cells (AGS cells) were selected as a model to evaluate the antioxidant capacity of *Prumnopitys andina* fruit extracts considering that this cell line has unaltered functional responses to oxidative stress, similar to that of non-tumor cells [35]. In addition, the use of epithelial cells from the gastrointestinal tract is appropriate since polyphenols may exert their protective effects before their absorption into the bloodstream [36,37].

**Figure 4:**
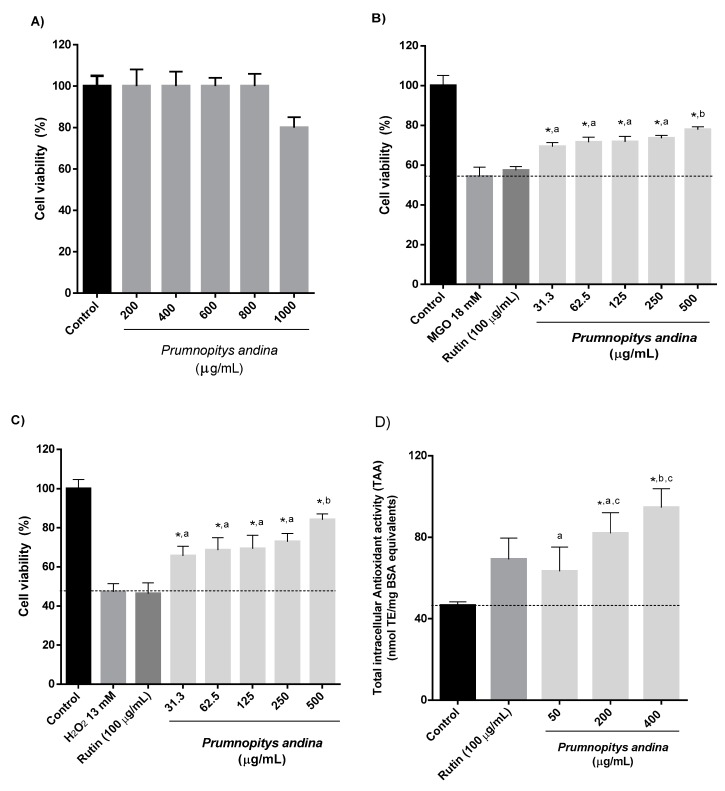
(**A**) Effect on the viability of human gastric epithelial cells (AGS) pre-treated with *Prumnopitys andina* fruit extract, (**B**) effect on the viability of AGS cells pre-treated with *P.* andina fruit extract and subsequently challenged with methylglyoxal (MGO), and (**C**) H_2_O_2_ (* *p* < 0.05 compared to damage controls, *n* = 5, two independent experiments); (**D**) Effect of the pre-incubation of AGS cells with *P. andina* fruit aril extract on the total intracellular antioxidant activity (TAA). Results are expressed as means ± SD (* *p* < 0.05 compared to untreated controls, *n* = 5, two independent experiments). Different letters (a–c) on each column indicate statistical differences according to Tukey’s test (*p* < 0.05).

Under the conditions of our assay, the pooled samples of *P. andina* fruit extract showed no cytotoxic effect towards AGS cells in concentrations up to 800 µg/mL (Figure 4A). In a study with human volunteers with ileostomy, after the consumption of 300 g of blueberries, about 85% of the polyphenol content reached the colon [38]. In another study, about 7.5% of polyphenols administered to rats were bioavailable in the small intestine [39]. Considering these data, and the normal consumption of fruits in the diet, concentrations ranging from 0.01–500 µg extract/mL can be considered appropriate for cellular-based assays when using epithelial gastrointestinal lines [40]. The flavonol rutin (100 µg/mL) was included in this assay for comparison purposes, considering that it was one of the main metabolites isolated from *P. andina* fruit extract.

In the cytoprotective assay against dicarbonyl-induced damage, the methylglyoxal (MGO) control presented a cell viability of 54.4%, while rutin showed a non-significant effect of 57.4% viability. A direct effect was observed for *P. andina* XAD7 extract (31.3–500 µg/mL) with significant cell viability percentages between 69.3–77.9% (Figure 4B). The Tukey’s test indicated no significant differences among the concentrations 31.3–250 µg/mL, while the concentration of 500 µg/mL was significantly different from the other concentrations assayed. 

Reactive oxygen species (ROS) are produced by many physiological processes, but can also be induced and stimulated by exogenous ROS-generating substances such as H_2_O_2_ [41]. Hydrogen peroxide is an important source of ROS in cells, due to its ability to cross cell membranes and to be converted into other free radicals, such as superoxide anion and hydroxyl radical [41]. In our experimental model, the H_2_O_2_ control presented a cell viability of 47.4%, while rutin showed a non-significant effect of 46.4% viability in the cytoprotective assay against oxidative-induced damage. A direct effect was observed for *P. andina* extract (31.3–500 µg/mL), with significant cell viability percentages between 65.7–84.2% (Figure 4C). The Tukey’s test again indicated no significant differences among the concentrations ranging from 31.3 to 250 µg/mL, while the concentration of 500 µg/mL was significantly different from the other concentrations assayed. In agreement with our results, glycosylated polyphenols such as rutin did not demonstrate cytoprotective effect against oxidative stress [42]. Caffeic acid has demonstrated cytoprotective effects against H_2_O_2_-induced cytotoxicity in Jurkat cells, but its activity was lower than more lipophilic compounds such as flavonols [43]. Hence, although rutin and caffeic acid β-glycoside are main components of the *P. andina* XAD7 fruit extract, they might contribute little to the observed activity. This means that other components of the extracts could be responsible for the reported observations. Due to the low amount of 20-hydroxyecdysone recovered after spectrometric analysis, it was not possible to include this compound in this assay to evaluate its contribution to the cell survival against oxidative and dicarbonyl-induced stress.

In the total intracellular antioxidant assay (TAA), the *P. andina* XAD7 extract showed a significant increase of 76.1% and 103.4% at concentrations of 200 and 400 µg/mL, respectively, compared to the untreated controls (Figure 4D). At 50 µg/mL, a non-significant increase of 35.9% was observed. The Tukey’s test indicated no significant differences between the concentration of 50 and 200 µg/mL, and between the concentrations of 200 and 400 µg/mL. Rutin induced a non-significant increase of 48.6%. Interestingly, the fruit extract from the Australian *Podocarpus elatus* (Illawarra plum) did not protect RAW 264.7 cells from H_2_O_2_-induced damage, at concentrations ranging from 31.2–500 µg/mL [9]. In another study, *P. elatus* fruit extracts lowered the expression of COX-2 and iNOS induced by LPS in RAW 264.7 cells, in a concentration dependent manner [44]. It needs to be considered that the data obtained is normalized by the total protein content measured by the BCA reaction. However, the methodology may underestimates the protein content, and thus overestimates the effect of the extract.

In summary, the *P. andina* XAD7 fruit extract showed cytoprotective effects against MGO and H_2_O_2_-induced stress, and increased the total intracellular antioxidant activity. Nevertheless, it needs to be considered that our observations are in vitro, showing only a potential activity of the *P. andina* fruit extracts. The cell-based methods do not fully take into account the physiological conditions of the gastrointestinal environment, such as the effect of detoxifying enzymes, the effects of the gastrointestinal digestion and microbiota on the composition of the extract, and the incubation period that does not mimic the gastric emptying time. Hence, the observed effects should be confirmed using in vivo models. 

## 3. Materials and Methods 

### 3.1. Reagents and Chemicals

The chemicals, enzymes and reagents acquired from Sigma-Aldrich (St. Louis, MO, USA) were the following: α-amylase from porcine pancreas (A3176; EC 3.2.1.1), α-glucosidase from *Saccharomyces cerevisiae* (G5003; EC 3.2.1.20), acetic acid, AlCl_3_, Amberlite XAD7 HP, catechin, DPPH (2,2-diphenyl-1-picrylhydrazyl radical), 3,5-dinitrosalicylic acid, porcine pancreas lipase type II (L-3126; EC 3.1.1.3), Na_2_CO_3_, 4-nitrophenyl-α-d-glucopyranoside, *p*-nitrophenyl palmitate, *p*-anisaldehyde, quercetin, sodium acetate, starch, 2,4,6-tri(2-pyridyl)1,3,5-triazine (TPTZ), trichloracetic acid, thiobarbituric acid, and triton X-100. The following materials were purchased from Merck (Darmstadt, Germany): 6-hydroxy-2,5,7,8-tetramethylchroman-2-carboxylic acid (Trolox), ethyl acetate, FeCl_3_ × 6H_2_O, potassium sodium tartrate and HPLC-grade methanol. Pepsin A (P3271) was from US Biological (Salem, MA, USA). Other buffer salts were purchased from JT Baker (Xalostoc, Mexico) and Scharlau Chemicals (Barcelona, Spain). The reference compound Orlistat was obtained from Laboratorio Chile (Santiago, Chile). A Barnsted EasyPure equipment (Thermo Scientific, Marietta, OH, USA) was used for the ultra-purification of water.

### 3.2. Plant Material

The ripe fruits from *Prumnopitys andina* (Poepp. et Endl. de Laub, Podocarpaceae) were collected in “El Melado”, Región del Maule (35°36′ S; 70°57′ W), Chile, during February 2016–2018, including two different samples from 2017. For the sample of 2016, a total of 131 g were collected (100 g of pulp). For the sample of 2017a, a total of 1.56 kg were collected (1.185 kg of pulp). For the sample of 2017b, a total of 553 g of fruit was processed (346.2 g of pulp). For the collection of 2018, a total of 2.488 kg were processed (1.833 kg of pulp). All the fruit material from each year and from the different collection places was pooled and extracted as detailed below. Voucher herbarium specimens have been deposited at the Herbario de la Universidad de Talca under code numbers FJ-PA-2016, FJ-PA-2017A, FJ-PA-2017B and FJ-PA-2018.

The fruits were transported to the laboratory and washed. The arils were then separated from the seeds by hand. Small amounts of fruit were extracted with different solvents to obtain the best yield of extraction of polyphenols (data not shown). This preliminary analysis indicated that the best solvent to obtain the maximum yield was MeOH (Table 1). Hence, the pulp was homogenized in a blender (Thomas TH-501V, Thomas Elektrogeräte, Shanghai, China) and extracted four times with MeOH in a 1:3 *v/v* homogenate:solvent ratio in the dark (10 min each). The extraction process was enhanced using an ultrasonic bath at room temperature and 35 kHz (Elma Transonic 700, Elma GmbH and Co. KG, Singen, Germany). Extracts were separately dried under reduced pressure at 37 °C in a rotary evaporator (R-200 Büchi, Flawil, Switzerland). The dried MeOH extract was resuspended in 1 L of water and sonicated 5 min to improve the solubility of the compounds. Compounds of interest in the extract (including genines and glucosides) were concentrated by adsorption with an Amberlite^®^ XAD resin. The solution was mixed with the resin in a 1:5 extract:Amberlite ratio. The mixture was stirred for 40 min, and the resin with the absorbed compounds was filtered through a filter paper and washed with 3 L of distilled water to ensure that the contaminants were removed. Finally, secondary metabolites were desorbed from the resin with 3 L of MeOH. The solvent was then evaporated in a rotary evaporator at 37 °C to dryness. The solid residue was then freeze-dried (Scanvac Coolsafe 55-15 Pro, Labogene, Allerød, Denmark). The resulting powder, from here forward referred to as the XAD7 extract, was the base material used in the analyses described below. 

### 3.3. Extract Fingerprinting by HPLC-DAD

HPLC-DAD analyses were performed on a Shimadzu Prominence equipment (Shimadzu Co., Kyoto, Japan), equipped with an LC-20AT pump, SPD-M20A UV diode array detector, and CTO-20AC column oven at 25 °C, and Labsolution software (Shimadzu Co.) Extract components were separated on a MultoHigh 100 C18 column, 5 µm, 250 × 4.6 mm (CS-Chromatographie Service GmbH, Langerwehe, Germany), eluted at a flow rate of 0.6 mL/min with the following gradient of acetonitrile (solvent A) and H_2_O-formic acid (99:1, *v/v*, solvent B). Initial conditions were 5% A and 95% B. A linear increase to 15% A, 85% B was carried out during 15 min and hold until min 20. Then, a linear increase to 18% A, 82% B was carried out during 5 more min. From min 25 to min 62, a linear increase up to 20% A, 80% B was performed. The system was linearly increased to yield 100% A, 0% B at min 65. The solvent system was returned to the initial condition of 5% A, 95% B with a linear decrease finalizing in min 75. The final conditions were maintained during 10 min to equilibrate the column for the next injection. The UV spectra from the chromatograms were recorded from 200 to 600 nm for peak characterization, and the elution of peaks was monitored at 280, 320 and 360 nm in parallel.

For quantification of main compounds, samples were injected three times, areas of peaks detected at 360 nm for rutin and 320 nm for caffeoyl glucoside were calculated, and compound concentrations were determined by comparison to six-point calibration curves for rutin (97.2% purity, R = 0.9999, limit of detection (LOD): 0.04 μg, limit of quantification (LOQ): 0.13 μg) and trans-caffeic acid (99.8% purity, R = 0.9997, LOD: 0.44 μg, LOQ: 1.34 μg). Results were expressed as mg/100g of extract.

### 3.4. Isolation and Characterization of Main Compounds from P. andina Fruit Extract

#### 3.4.1. Sephadex LH-20 Permeation/Column Chromatography

Sephadex permeation is a traditional strategy to partition a complex extract into more simple fractions according to their molecular weight and polarity. The XAD7-extract was loaded on to a Sephadex LH-20 column (length 103 cm, internal diameter 5 cm, Sephadex height 38 cm) and eluted first with a mixture of MeOH:H_2_O (1:1, 2 L), and then with MeOH (1L). The experiment was repeated twice using first 2.7 g of XAD7 extract and 3.16 g the second time. The void volume was 187 mL. Forty fractions of 80 mL each were collected and solvent was reduced to about 10 mL in each fraction under reduced pressure. Molecular components of each fraction were then separated on thin layer chromatographic (TLC) on TLC ALUGRAM^®^ plates (Macherey-Nagel GmbH & Co, Düren, Germany) developed with ethyl acetate:acetic acid:water 10:2:3 *v/v/v*, with *p*-anisaldehyde visualization at 65 °C. Fractions with similar TLC patterns were pooled as follows: 1–6, 7–8, 9–12, 13–14, 15–18, 19–20, 21–22, 23–24, 25–26, 27–28, 29–30 and 31–32. Fractions showing single compounds by TLC were analyzed by ^1^H- and ^13^C-NMR and Q-TOF mass spectrometry for structural elucidation.

#### 3.4.2. Counter-Current Chromatography (CCC) Separation

Counter-current chromatography (CCC) is an automated version of liquid-liquid extraction based on the partition of an analyte between two immiscible phases. The biphasic solvent system can be adjusted according to the polarity of the target compounds to tailor separations [11]. As an adjunct to standard column separation methods, CCC offers several advantages, including high sample recovery, high purity of fractions and large sample loading capacity, and it eliminates the risk of irreversible adsorption of the extract to the stationary phase [12].

The choice of the correct biphasic solvent system is the critical step in any CCC separation [12]. Several solvent systems of different polarities need to be evaluated to determine the partition coefficients (K*_D_*). The K*_D_* values need to be within the “sweet-spot”, which means, values between 0.5 < K*_D_* < 2.5 [12]. If the targeted compounds show K*_D_* values within this range, and a resolution factor of at least 1.5, a successful separation is almost guaranteed [12]. To obtain proper partition coefficients, several mixtures of tert-butylmethylether-butanol-acetonitrile-water (TBME/BuOH/ACN/H_2_O) were prepared [12]. Ten mg of the extract were dissolved in 4 mL of each equilibrated solvent system (1:1, *v/v*). After shaking, the mixture was left to re-equilibrate into a biphasic system. Using a micropipette, 1 mL of each phase was withdrawn, and components were separated by TLC as described above. For partitioned systems showing the presence of compounds in both phases, phases were separated and solvents were removed. Then, components in each phase were separated by HPLC-DAD and the area under the curve (AUC) of each chromatographic peak was quantitated. The K_D_ values were calculated considering the organic upper phase (UP) as stationary and the aqueous lower phase (LP) as mobile phase (*head-to-tail* mode) using the following formula:

(1) $$K_{D}=\frac{AUC\;UP}{AUC\;LP}$$

Following the K*_D_* value analyses, the solvent system showing the most compounds within the “sweet-spot” was selected. 

The CCC separation was performed on a *J*-type Quattro MK5 Lab Prep (AECS, Wales, UK), equipped with four polytetrafluoroethylene (PTFE) coils (5 × 2.16mm), total column volume: 500 mL, revolution radius: 120 mm, and rotation speed: 650 rpm. A 10 mL external loop was used to manually inject the sample into the column. The day of the experiment, 2 L of the selected solvent system were prepared, and the phases were separated using a 2 L glass separatory funnel. The upper and lower phases were degassed by sonication at room temperature. The CCC system was first filled with the organic stationary phase using a HPLC-pump (Series II, Scientific Systems Inc., State College, PA, USA). Then, rotation was set to 650 rpm and the mobile aqueous phase was pumped at 4.0 mL/min. When the hydrodynamic equilibrium was reached, the sample was dissolved in 10 mL of a 1:1 mixture of both phases, sonicated and filtered in a Clarinert Syringe filter (Agela Technologies, Torrance, CA, USA). The sample was then injected into the CCC using a 10 mL external loop. The temperature of the equipment was maintained at 21 °C during the run. Fractions were collected with a Gilson FC 203B (Middleton, WI, USA), set at 2 min/tube and pooled after TLC comparison in the same system described above. A total of 90 fractions were collected, and then rotation was stopped for the extrusion step using MeOH:H_2_O (6:4) at 5 mL/min until 600 mL were collected. The fractions were pooled as follows: 1–16, 17–19, 20–21, 22–23, 24, 25, 26–30, 31–39, 40–49 and 50–90. Fractions showing single compounds by TLC analysis, were analyzed by ^1^H- and ^13^C-NMR, and by Q-TOF mass spectrometry.

### 3.5. Instrumental Analysis

NMR spectra including ^1^H, ^13^C, distortionless enhancement by polarization transfer (DEPT) 135, heteronuclear multiple bond correlation (HMQC) and heteronuclear multiple quantum correlation (HMBC) experiments were measured in CD_3_OD using a Bruker Avance 400 (Bruker Biospin, Rheinstetten, Germany) spectrometer at 400 MHz for ^1^H and 100 MHz for ^13^C. The chemical shifts were calibrated against the residual solvent signals and are given in ppm. Coupling constants are given in Hz. Offline data processing was carried out using the Mnova software package (Mestrelab Research, S.L., Santiago de Compostela, Spain).

ESI-MSn analyses were conducted in a Micromass Q-TOF instrument (Manchester, UK). Fractions were directly infused at a flow rate of 10 µL/min using a syringe pump (Harvard Apparatus, Holliston, MA, USA). Mass spectra and tandem mass spectra were acquired in the negative ion mode. Operation conditions were: 3.0 kV capillary voltage, 40 V cone voltage and 100 °C for the desolvation gas. Tandem mass spectra were induced by collision dissociation (CID) of the mass selected deprotonated molecules, using Ar as the buffer gas and collision energies of 5–45 eV. Mass selection was carried out by quadrupole 1 using a unitary m/z window, and collisions were performed in the rf-only quadrupole collision cell followed by time of flight (TOF) mass analysis in the range of m/z 100–1000 amu. Infrared (IR) spectra of isolated compounds were acquired on a Nexus Nicolet 470 Fourier-transform IR (FT-IR) transmission spectrophotometer system (Thermo Nicolet Corp, Madison, WI, USA). Optical rotations were determined in MeOH using a Jasco DIP-370 digital polarimeter (Jasco Spectroscopic Co. Ltd. a, Tokyo, Japan).

### 3.6. HPLC-DAD-MS/MS Analysis

The HPLC analyses were carried out in an Agilent Series 1100 HPLC system equipped with a G1311 quaternary pump, a G1315B diode array detector, a G1322A degasser, G1313A autosampler, and a LC/MSD Trap VL G-2445 electrospray ionization mass spectrometry (ESI-MS^n^) detector. The data analysis was performed using the ChemStation software (Agilent Technologies, Waldbronn, Germany). A Zorbax Eclipse XDB C18 column (3.5μm, 150 × 2.1 mm) (Agilent, Germany) was used for the separation of compounds. The solvent systems were: A (H_2_O-formic acid-ACN, 88.5:8.5:3, *v/v/v*); B (H_2_O-formic acid-ACN; 41.5:8.5:50, *v/v/v*); and C (H_2_O-formic acid-MeOH, 1.5:8.5:90, *v/v/v*,). The analysis was carried out using the following gradient: t = 0 min, 98%A, 2%B and 0%C; t = 8 min, 96%A, 4%B and 0% C; t = 37 min, 70%A, 17%B and 13%C; t = 51 min, 50%A, 30%B and 20%C; t = 51.5 min, 30%A, 40%B and 30% C, t = 56 min, 0%A, 50%B and 50%C; t = 57 min, 0%A, 50%B and 50%C; t = 64 min, 98%A 2%B and 0%C. A flow rate of 0.19 mL/min was used and temperature was set at 40 °C, with an equilibration time of 8 min in the initial conditions before the next injection. The ESI-MS^n^ analysis was carried out in the negative mode, using nitrogen as the nebulizer gas at 40 psi, 350 °C and at a flow rate of 8 L/min. Other parameters were: electrospray needle, 3500 V; skimmer 1, 20.3 V; skimmer 2, 6.0 V; capillary exit offset 1, 68.2 V; capillary exit offset 2, 88.5 V. The scan mode was performed at a speed of 13,000 *m/z*/s, in the range of 50–1000 *m/z*.

### 3.7. Total Phenolic and Total Flavonoid Content

The total content of phenolics (TP) in the XAD7 fruit extracts was spectrophotometrically determined using the Folin-Ciocalteu method [45]. The use of the XAD7 extracts avoids the interference of other reducing substances, allowing an accurate determination of the TP content. Gallic acid was used for the calibration curve. The results are expressed as gallic acid equivalents (GAE)/100 g XAD7 extract. The total content of flavonoids (TF) was determined according to Jiménez-Aspee et al. [24]. Briefly, XAD7 samples were dissolved in MeOH at a concentration of 3 mg/mL. A 250 µL aliquot was mixed with 75 µL 5% NaNO_2_, mixed and then left to stand for 5 min. Then, a 100 µL aliquot of 10% AlCl_3_ solution was added, mixed and left to stand for 5 min. Finally, a 500 µL aliquot of 1 M NaOH was added and volume was completed to 5 mL using distilled water. After 30 min, the absorbance was read in a spectrophotometer (Genesys 10uv, ThermoSpectronic, Waltham, MA, USA) at 510 nm. Catechin was used as for the calibration curve and results are expressed as catechin equivalents (CAE)/100 g XAD7 extract. All determinations were carried out in triplicate and results are presented as mean values ± standard deviation (SD).

### 3.8. Antioxidant Activity

The antioxidant capacity of the extracts was evaluated by the discoloration of the DPPH radical, the ferric reducing power (FRAP), the scavenging of the ABTS radical (TEAC) and the oxygen radical absorbance capacity (ORAC). The XAD7 fruit extracts were dissolved at an initial concentration of 300 μg/mL. Trolox and quercetin were used as reference compounds. The DPPH, FRAP and TEAC assays were performed according to Jiménez-Aspee et al. [25]. The ORAC assay was carried out following the methodology of Ou et al. [46] with slight modifications [47]. All determinations were carried out in triplicate and results are presented as mean values ± standard deviation (SD).

### 3.9. Inhibition of Carbohydrate- and Lipid-Metabolizing Enzymes 

#### 3.9.1. α-Glucosidase 

Potential inhibition of α-glucosidase by XAD7 extracts and fractions from the LH-20 column was tested under conditions that simulate, at least in part, those of the small intestine [48]. The reaction mixture contained 20 µL of α-glucosidase solution (0.25 U/L) and 120 µL of the extracts (100–0.1 μg/mL), previously dissolved in 200 mM sodium phosphate buffer (pH 6.8). The reaction mixture was incubated at 37 °C for 15 min to allow enzyme modification. Resulting enzyme activity was then measured by adding 20 µL of 5 mM *p*-nitrophenyl-α-D-glucopyranoside into the wells and incubating for additional 15 min at 37 °C. Reaction was halted by adding 80 µL of 0.2 M Na_2_CO_3_. Final absorbance was measured at 415 nm in a Biotek ELx801 (Winooski, VT, USA) microplate reader. Results are reported as IC_50_ values in μg extract/mL using acarbose, a known α-glucosidase inhibitor, as the standard for full inhibition and reaction without the extract as the standard for full reaction. All samples were assayed in triplicate. 

#### 3.9.2. α-Amylase

Potential inhibition of α-amylase by XAD7 extracts was tested using the conditions described by Bernfeld [49], with slight modifications. A 100 µL aliquot of the samples (100–10 μg/mL in 20 mM phosphate buffer, pH 6.9 + 6.7 mM NaCl) was mixed with 100 µL of 1% *w/v* starch in phosphate buffer and 100 µL of the phosphate buffer. The mixture was heated at 37 °C for 5 min to swell and gelatinize the starch. Then, 100 µL of α-amylase solution (8 U/mL in ice cold water, pH 5.5) was added and incubated for 20 min at 37 °C. The colour reagent was prepared by mixing 20 mL of 96 mM 3,5-dinitrosalicylic acid with 8 mL of 5.31 M sodium potassium tartrate in 2 M NaOH and 12 mL of distilled water. An aliquot of 200 µL of this solution was added to the reaction mixture and boiled for 15 min in tightly closed Eppendorf tubes to inactivate the enzyme and avoid changes in volume by evaporation. An aliquot of 40 µL of each tube was mixed with 210 µL of distilled water and placed in a 96-well microplate. The final absorbance was read at 550 nm in a microplate reader. Results are reported as IC_50_ values in μg extract/mL using acarbose, a known α-amylase inhibitor, as the standard for full inhibition; and reaction without the extract as the standard for full reaction. All samples were assayed in triplicate.

#### 3.9.3. Lipase 

Potential inhibition of pancreatic lipase by XAD7 extracts was tested as described by McDougall et al. [28], with slight modifications. The enzyme, pancreatic porcine lipase type II, was suspended in ultrapure water at 20 mg/mL and allowed to hydrate for 6 min at 4 °C. Then, the mixture was centrifuged at 20,000× *g* for 10 min at 4 °C. The resulting supernatant was used for the assay. A *p*-nitrophenyl palmitate solution, the substrate, was prepared at 0.08% *w/v* in 5mM sodium acetate buffer (pH 5.0), supplemented with 1% Triton X-100. The mixture was heated in boiling water for 2 min to increase the dissolution, and then allowed to cool down to room temperature before use. The assay mixture contained 50 µL of the extract (50–10 μg/mL in distilled water), 150 µL of the enzyme, 450 µL of substrate and 400 µL of the assay buffer (100 mM Tris, pH 8.2). The mixture was incubated without shaking at 37 °C for 2 h. At the end of the incubation, absorbance was determined at 400 nm in an ultraviolet–visible (UV–Vis) spectrophotometer (Genesys 10uv, ThermoSpectronic, Waltham, MA, USA). Results are reported as IC_50_ values in μg extract/mL using orlistat, the commercial pancreatic lipase inhibitor, as the standard for full inhibition; and reaction without the extract as the standard for full reaction. Six replicates were assayed for all samples.

### 3.10. Determination of Thiobarbituric Acid Reactive Substances (TBARS) in a Simulated Gastric Digestion Model

This assay was carried out according to Kanner et al. [30], with slight modifications. Briefly, the simulated gastric fluid (SGF) contained final concentration of NaCl (0.2% *w/v*), pepsin (0.32% *w/v*) and 700 µL of HCl (37%), adjusted to a final volume of 100 mL with ultrapure water. Thirty grams of turkey meat were ground with 90 mL of the SGF solution for 1 min in a blender and adjusted to pH 3.0 with 100 µL of 7N HCl. Fifteen mL of the SGF-meat mixture was aliquoted in 100 mL Erlenmeyer flasks. The powdered extract was weighed and added to the homogenate (meat–liquid mixture) to achieve final concentrations ranging from 0–500 µg/mL in the 15 mL SGF-meat mixture. The *P. andina* sample consisted in a pool of the extracts from different collection years. The flasks were placed in a shaking water bath at 37 °C during 1 h. At the end of the incubation, 800 µL of the samples, controls (without extracts) and blanks (without meat) were centrifuged at 20,000× *g* for 15 min at 4 °C. Lipid peroxidation was determined quantifying TBARS. For this purpose, the supernatant resulting from the centrifugation (750 µL) was mixed with 750 µL of TBA solution (0.37% TBA, 15% trichloroacetic acid in 0.25 N HCl), and boiled during 20 min. The absorbance was determined at 532 nm. All determinations were carried out in triplicate and results are presented as IC_50_ values (μg extracts/mL) ±SD. Aminoguanidine, a known nucleophile that reacts with electrophiles arising from lipid peroxidation [50], was used as the standard compound for inhibition; and the reaction without the extract was used as the standard for full reaction. All samples were assayed in triplicate.

### 3.11. Cell-Based Experiments

#### 3.11.1. Cell Culture 

Human AGS gastric adenocarcinoma cells (ATCC CRL-1739, American Type Culture Collection, Manassas, VA, USA) were grown as monolayers in a humidified incubator with 5% CO_2_ in air at 37 °C in HamF-12 medium supplemented with 2 mM L-glutamine and 1.5 g/L sodium bicarbonate. The medium contained 10% heat inactivated fetal bovine serum (FBS), 100 IU/mL penicillin and 100 µg/mL streptomycin. Cells were plated at 25,000 cells/mL in 96-well plates, with 100 µL per well. Cells were counted by means of the trypan blue exclusion assay in a Neubauer chamber. All the cell-based experiments were carried out using a pool of the extracts from different collection years.

#### 3.11.2. Determination of the Cytotoxicity of *P. andina* XAD7 Fruit Extracts in Human Gastric Epithelial Cells (AGS)

The cytotoxicity values, expressed as IC_50_ in µg extract/mL, were determined in a first step to establish the working concentrations in the subsequent experiments. Confluent cultures of AGS cells were treated during 24 h with medium containing the pool of *P. andina* XAD7 fruit extracts at concentrations ranging from 0–1000 µg/mL. The sample was dissolved in complete medium supplemented with 2% FBS. At the end of the incubation period, 100 µL of a 0.5 mg/mL MTT solution (in medium only) was added to each well and plates were incubated for 4 h at 37 °C and 5% CO_2_. Then, the MTT solution was discarded and 100 µL of ethanol acidified with 0.5% HCl was added to each well. Plates were shaken for 10 min in an orbital shaker and finally absorbance was measured at 550 nm in a microplate reader [35]. The 100% viability control was determined using cells treated with culture medium alone. Experiments were repeated twice using different cell preparations and concentrations were tested in quadruplicate.

#### 3.11.3. Cytoprotection Against H_2_O_2_ or Methylglyoxal (MGO)-Induced Stress 

Confluent cultures of AGS cells were incubated for 16 h with 100 µL of the different samples at concentrations ranging from 0 to 400 µg extract/mL). The flavonol rutin (100 µg/mL) was included in this assay for comparison purposes. The sample was dissolved in medium supplemented with 2% FBS and antibiotics. Culture medium was completely removed by vacuum aspiration at the end of the incubation, and cells were washed with 100 µL of PBS. Then, stress was induced using 100 µL of 13 mM H_2_O_2_ or 18 mM MGO for 20 min and 35 min, respectively [25]. The H_2_O_2_ and MGO solutions were freshly prepared using medium without supplements. Untreated cells were used as 100% viability controls. Cells treated with H_2_O_2_ or MGO served as damage controls. Cell viability was determined by means of the MTT reduction assay, as described above [35]. The experiments were repeated twice. Each concentration of the assayed samples was evaluated in quintuplicate. Results are expressed as percentage of the 100% viability control.

#### 3.11.4. Intracellular Total Antioxidant Activity (TAA)

The TAA was carried out according to Ferrari et al. [51]. AGS cells were plated at 25,000 cells/mL in 3 cm diameter Petri dishes. One day after confluence, AGS cells were pre-incubated overnight (16 h) with 3 mL of the pool of *P. andina* XAD7 extracts, in different concentrations (0–400 µg extract/mL). For comparison purposes, rutin (100 µg/mL) was also included in this experiment. All samples were prepared in medium supplemented with 2% FBS. Cells treated only with culture medium were used as the control of the basal antioxidant level. The next morning, cells were collected with a cell scraper and centrifuged at 900× *g* during 5 min. The pellet was re-suspended in 600 µL of cold PBS and lysed using a Branson SFX250 Sonifier, equipped with a 102-C converter and a double-step 1/8″ microtip with coupler, at a frequency of 20 kHz (Branson Ultrasonics Corp., Danbury, CT, USA). The sonication process was carried out during 12 s on an ice-water bath. The cell lysate was centrifuged (8000× *g*, 15 min, 4 °C) and the supernatant was collected and stored at −80 °C until the determination (within 3 weeks). 

The ABTS radical was prepared by mixing 5 mL of a 7.46 mM ABTS solution with 88 μL of a 140 mM potassium persulfate solution. The solution was stored 16 h in the dark at room temperature. At the end of the incubation, the solution was diluted with MeOH until an absorbance value of 0.700 ± 0.050 at 734 nm was reached. Cell lysates (30 µL) were mixed with 2.87 mL of the ABTS^+·^ radical. After 6 min, absorbance was measured at 734 nm in a UV–Vis spectrophotometer. The absorbance was interpolated and converted to Trolox equivalents using a Trolox calibration curve. The results were normalized to the total protein content, by means of a Pierce™ BCA Protein Assay Kit (23225, ThermoScientific, Rockford, IL, USA), using bovine serum albumin as standard protein. All the determinations were carried out in triplicate and results were expressed as µmol Trolox equivalents/mg protein ±SD.

### 3.12. Statistical Analyses

Analysis of variance (ANOVA-one way) followed by Tukey’s test was used to determine significant differences (*p* < 0.05). ANOVA (one way) followed by Dunnett’s multiple comparison test was used to assess statistical differences between cell-based experiments and their respective controls (*p* < 0.05). Significant differences among the concentrations evaluated in the cell-based experiments were determined by ANOVA followed by Tukey’s multiple comparison test (*p* < 0.05). The software SPSS 14.0 (IBM, Armonk, NY, USA) and the statistical package Statistica 13.0 (Dell Inc., Tulsa, OK, USA) were used for statistical analyses.

## 4. Conclusions

A total of 24 compounds were detected in the fruit extract from *P. andina*. Rutin, caffeic acid β-glucoside and 20-hydroxyecdysone were isolated by means of Sephadex and countercurrent chromatographies. Orientin, rutin, quercetin acetylglucoside and 3-caffeoylquinic acid were identified with commercial standards. The antioxidant activity determined by chemical methods was low, comparable to other Podocarpaceae fruits. Interestingly, in the cell-based assays, the fruit extract significantly protected AGS cells against oxidative and dicarbonyl stress in a dose-dependent manner. Moreover, the *P. andina* extract significantly increased the total intracellular antioxidant activity in gastric epithelial cells. These observations are a first approach into understanding the potential beneficial effects of *P. andina* fruit extracts. However, more studies are needed, including studies with animals and humans, in order to validate our findings. Hence, the results obtained in the in vitro assays for *P. andina* fruits suggest potential for biological activity and encourage more detailed research on its chemistry. Overall, the results of this study add relevance to the traditional use of *P. andina* fruits as a healthy complement in diets and support conservation of existing stocks and the domestication of this species.

## Figures and Tables

**Table 1:** ^1^H and ^13^C nuclear magnetic resonance (NMR) data of 20-hydroxyecdysone (20-HE) and its triacetate (20-HEAc) isolated from *P. andina* fruit extract.

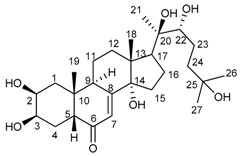 20-HE			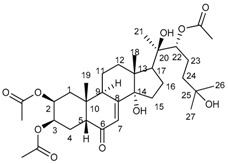 20-HEAc		
H	20-HE	20-HEAc	C	20-HE	20-HEAc
1	1.33 m, 1.68 m	1.50 m, 1.85 m	1	35.97 t	34.02 t
2	3.87 dt (12.0, 3.2)	5.03 dt (12.4, 3.2)	2	67.31 d	68.61 d
3	3.97 br s	5.33 br s	3	67.12 d	67.00 d
4	1.62–1.64 m	1.55 m, 2.03 m	4	31.44 t	31.73 t
5	2.40 dd (12.0, 4.4)	2.36 dd	5	50.38 d	50.95 d
6			6	205.06 s	201.99 s
7	5.83 br s	5.84 sbr	7	120.74 d	121.63 d
8			8	166.59 s	164.39 s
9	3.17 dd br (8.4, 8.4)	3.08 t (7.6)	9	33.70 d	33.56 d
10			10	37.87 s	38.39 s
11	1.68 m, 1.86 m	1.63 m, 1.77 m	11	20.10 t	20.53 t
12	1.76 m, 2.14 ddd (12.8, 8.0, 4.4)	1.83 m, 2.08 m	12	31.11 t	31.04 t
13			13	48.03 s	47.49 s
14			14	83.84 s	84.57 s
15	1.49 m, 1.87 m	1.68 m, 1.78 m	15	30.39 t	29.24 t
16	1.17 m, 1.55 m	1.45 m, 1.71 m	16	25.94 t	24.79 t
17	2.39 m	2.35 m	17	49.13 d	49.50 d
18	0.91 s	0.82 s	18	16.66 q	17.46 q
19	0.99 s	1.00 s	19	23.02 q	23.81 q
20			20	76.53 s	77.34 s
21	1.22 s	1.19	21	19.67 q	
22	3.35 d (13.6)	4.82 d (9.2)	22	77.02 d	79.63 d
23	1.6–1.7 m	1.6–1.7 m	23	20.10 t	20.36 t
24	1.34 m, 1.70 m	1.38–1.48 m	24	40.98 t	40.27 t
25			25	69.92 s	70.60 s
26	1.22 s	1.21 s	26	28.31 q	30.06 q
27	1.21 s	1.20 s	27	27.59 q	28.85 q
Acetate			Acetate		
			C=O		172.49 s, 170.48 s, 170.20 s
CH_3_		2.08 s, 2.08 s, 1.98 s	CH_3_		21.14 q (2C), 21.10 q

**Table 2:** Tentative identification of secondary metabolites of *P. andina* fruit extract by HPLC-DAD-electrospray ionization (ESI)-MS^n^.

Peak	Rt [min]	λ_max_ [nm]	[M−H]^−^	MS^2^	Tentative I Dentification
**1**	4.6	326, 300sh, 285	341.01	178.82 (91.4), 161.0 (38.9), 143.0 (13.4)	Caffeic acid β-glucoside 1 *,^#^
**2**	19.2	280	299.13	136.80 (100)	Hydroxybenzoic acid hexoside
**3**	21.3	-	315.20	152.84 (100), 108.8 (4)	Dihydroxybenzoic acid hexoside
**4**	23.8	326. 292 sh	514.99	352.66 (100). 190.70 (66)	3,5-dicaffeoylquinic acid
**5**	27.1	321, 285	515.21	352.80 (32). 341.90 (24) 323.00 (100). 190.99 (32). 178.90 (8)	Dicaffeoylquinic acid
**6**	30.8	330, 277	497.39	334.93 (100), 178.90 (10)	Caffeoylshikimic acid hexoside
**7**	34.1	321, 288	341.28	178.89 (100), 135.00 (7)	Caffeic acid hexoside 2
**8**	35.6	325, 300sh	352.99	190.91 (100), 179.3 (0.4)	3-caffeoylquinic acid **
**9**	36.2	320sh, 285	449.30	286.94 (100). 269.0 (39). 259.0 (45)	Dihydrokaempferol hexoside
**10**	39.3	312, 292sh	337.08	190.89 (100), 163.5 (2)	5-*p*-coumaroylquinic acid
**11**	42.1	332sh, 294	465.03	338.95 (24), 284.94 (100). 150.91 (36)	Taxifolin hexoside
**12**	46.1	249	479.37	319.90, 159.30	20-Hydroxyecdysone *
**13**	46.3	352, 248	447.28	356.90 (44), 326.97 (100). 287.00 (29). 259.10 (18)	Orientin **
**14**	49.7	354, 248	609.14	446.97 (100), 285.0 (10)	Kaempferol dihexoside
**15**	52.0	320, 248	355.38	192.90 (100)	Hydroxymethoxycinnamic acid hexoside
**16**	57.4	344, 248	431.08	310.99 (100)	Apigenin-C-hexoside
**17**	58.9	353, 280	609.30	342.87 (6). 300.85 (100)	Quercetin rutinoside *,**,^#^
**18**	65.8	-	447.28	284.90 (100)	Kaempferol hexoside
**19**	68.2	341, 325, 280	463.23	343.00 (1.3). 300.88 (100)	Quercetin hexoside
**20**	68.6	350, 300sh	433.18	300.88 (100)	Quercetin pentoside
**21**	69.1	350, 297 sh	593.34	284.95 (100)	Kaempferol rutinoside
**22**	70.0	354, 280sh	623.29	315.02 (100)	Isorhamnetin rutinoside
**23**	70.2	-	505.54	462.98 (32). 300.91 (100)	Quercetin acetyl glucoside **
**24**	72.1	-	519.36	314.92 (100)	Isorhamnetin acetylhexoside

Retention time is according to Figure 3. * Identity confirmed by NMR analysis. ** Identity confirmed by co-injection with standard. ^#^ Compound quantified using external calibration curve-not determined.

**Table 3:** Yield of XAD7 extraction (%, *w/fw*), total phenolic content (TP), total flavonoid content (TF), antioxidant activity and inhibition of α-glucosidase by *P. andina* fruit extracts (*n* = 3).

Samples	Yield of Extraction (%)	TP (g GAE/100g XAD7 Extract)	TF (g CE/100g XAD7 Extract)	DPPH (SC_50_, µg XAD7 Extract/mL)	FRAP (µmol TE/g XAD7 Extract)	TEAC (µM TE/g XAD7 Extract)	ORAC (µmol TE/g XAD7 Extract)
2016	nd	4.1 ± 0.1 ^a^	2.4 ± 0.1 ^a^	93.6 ± 2.9 ^a^	183.0 ± 5.0 ^a^	206.8 ± 11.2 ^a^	859.7 ± 28.9 ^a^
2017a	1.1	2.0 ± 0.0 ^b^	1.3 ± 0.0 ^b^	>100	inactive	inactive	371.2 ± 17.3 ^b^
2017b	0.4	7.1 ± 0.1 ^c^	5.3 ± 0.1 ^c^	33.1 ± 0.4 ^b^	359.8 ± 10.3 ^b^	inactive	867.4 ± 48.3 ^a^
2018	0.3	7.3 ± 0.1 ^c^	5.0 ± 0.1 ^d^	67.6 ± 1.9 ^c^	473.3 ± 8.8 ^c^	514.7 ± 10.6 ^b^	1921.2 ± 149.8 ^c^
Quercetin *	-	-	-	7.8 ± 0.3	1077.2 ± 16.4	8157.9 ± 22.1	22561.6 ± 808.8

GAE: gallic acid equivalents; CE: catechin equivalents; TE: Trolox equivalents; * positive controls: -: not determined. All determinations were carried out in triplicate and results are expressed as mean values ± SD. Different letters (a–d) on each column show significant differences among each determination, according to Tukey’s test (*p* < 0.05).

**Table 4:** Inhibition of α-glucosidase, α-amylase and pancreatic lipase by *P. andina* fruit extracts (*n* = 3).

Samples	α-Glucosidase (IC_50_, µg XAD7 Extract/mL)	α-Amylase (IC_50_, µg XAD7 Extract/mL)	Pancreatic Lipase (IC_50_, µg XAD7 Extract/mL)
2016	8.7 ± 0.3 ^a^	inactive	inactive
2017a	4.8 ± 0.4 ^b^	inactive	inactive
2017b	4.6 ± 0.1 ^b^	inactive	inactive
2018	5.7 ± 0.2 ^c^	inactive	inactive
Acarbose *	137.7 ± 1.3 µg/mL	28.5 ± 0.3 µg/mL	ND
Orlistat	ND	ND	0.04 ± 0.00 µg/mL

ND: not determined; * positive controls; Different letters (a–c) on each column show significant differences among each determination, according to Tukey’s test (*p* < 0.05).

**Table 5:** Inhibition of α-glucosidase by the fractions obtained by Sephadex chromatography of the. *P. andina* fruit extract (*n* = 3).

Fraction	α-Glucosidase Inhibition	α-Amylase Inhibiton	Pancreatic Lipase Inhibition
1–6	39.6 ± 2.5%	inactive	inactive
7–8 (20-OH-ecdysone)	40.1 ± 1.3%	inactive	inactive
9–12	28.8 ± 2.1 µg/mL	inactive	inactive
13–14	3.4 ± 0.1 µg/mL	inactive	inactive
15–18	4.7 ± 0.2 µg/mL	inactive	inactive
19–20	2.9 ± 0.1 µg/mL	inactive	inactive
21–22 (Caffeic acid)	11.4 ± 0.7 µg/mL	inactive	inactive
23–24	9.9 ± 0.3 µg/mL	inactive	inactive
25–26	7.8 ± 0.2 µg/mL	inactive	inactive
27–28	8.4 ± 0.5 µg/mL	inactive	inactive
29–30	37.3 ± 1.1 µg/mL	inactive	inactive
31–32 (Rutin)	44.8 ± 1.6%	inactive	inactive

Percentages of inhibition were determined at a concentration of 50 µg/mL.

## Abbreviations

20-HE	20 hydroxyecdysone
AAPH	2,2′-azobis(2-amidinopropane) dihydrochloride
ABTS	2,2′-azino-bis(3-ethylbenzothiazoline-6-sulphonic acid)
ACN	acetonitrile
AGS	adenocarcinoma gastric epithelial cells
amu	atomic mass units
AUC	area under the curve
BuOH	butanol
CCC	counter-current chromatography
CE	catechin equivalents
COX	cyclooxygenase
DAD	diode-array detector
DEPT	Distortionless Enhancement by Polarization Transfer
DPPH	2,2-Diphenyl-1-picrylhydrazyl
FBS	fetal bovine serum
FRAP	ferric reducing antioxidant power
GAE	gallic acid equivalents
HPLC	high-performance liquid chromatography
HMBC	Heteronuclear Multiple Bond Correlation
HMQC	Heteronuclear Multiple-Quantum Correlation
iNOS	inducible nitric oxide synthase
LP	lower phase
LPS	lipopolysaccharide
MDA	malondialdehyde
MGO	methylglyoxal
MS	mass spectrometry
MTT	3-(4,5-dimethylthiazol-2-yl)-2,5-diphenyltetrazolium bromide
NMR	nuclear magnetic resonance
PBS	phosphate buffer saline
QTOF	quadrupole time of flight
ORAC	oxygen radical absorbance capacity
SGF	simulated gastric fluid
TAA	total intracellular antioxidant activity
TBARS	thiobarbituric acid reactive species
TE	trolox equivalents
TEAC	trolox equivalent antioxidant capacity
TF	total flavonoid content
TLC	thin layer chromatography
TP	total phenolic content
UP	upper phase
